# Deep Bayesian-Assisted Keypoint Detection for Pose Estimation in Assembly Automation

**DOI:** 10.3390/s23136107

**Published:** 2023-07-02

**Authors:** Debo Shi, Alireza Rahimpour, Amin Ghafourian, Mohammad Mahdi Naddaf Shargh, Devesh Upadhyay, Ty A. Lasky, Iman Soltani

**Affiliations:** 1 Department of Electrical and Computer Engineering, University of California Davis, Davis, CA 95616, USA; deshi@ucdavis.edu; 2 Greenfield Labs, Ford Motor Company, Palo Alto, CA 94304, USA; 3 Department of Mechanical and Aerospace Engineering, University of California Davis, Davis, CA 95616, USA

**Keywords:** keypoint detection, pose estimation, assembly automation, manufacturing automation, deep learning, AI, convolutional neural networks, robotics, robot manipulation

## Abstract

Pose estimation is crucial for automating assembly tasks, yet achieving sufficient accuracy for assembly automation remains challenging and part-specific. This paper presents a novel, streamlined approach to pose estimation that facilitates automation of assembly tasks. Our proposed method employs deep learning on a limited number of annotated images to identify a set of keypoints on the parts of interest. To compensate for network shortcomings and enhance accuracy we incorporated a Bayesian updating stage that leverages our detailed knowledge of the assembly part design. This Bayesian updating step refines the network output, significantly improving pose estimation accuracy. For this purpose, we utilized a subset of network-generated keypoint positions with higher quality as measurements, while for the remaining keypoints, the network outputs only serve as priors. The geometry data aid in constructing likelihood functions, which in turn result in enhanced posterior distributions of keypoint pixel positions. We then employed the maximum a posteriori (MAP) estimates of keypoint locations to obtain a final pose, allowing for an update to the nominal assembly trajectory. We evaluated our method on a 14-point snap-fit dash trim assembly for a Ford Mustang dashboard, demonstrating promising results. Our approach does not require tailoring to new applications, nor does it rely on extensive machine learning expertise or large amounts of training data. This makes our method a scalable and adaptable solution for the production floors.

## 1. Introduction

Despite significant advances in automation technology, the majority of assembly tasks across various industries are still carried out manually due to ongoing challenges related to the accessibility and ease of adoption. In addition to technological obstacles, the financial aspects of automation have not been favorable. The return on investment from automating a task on the production floor must be realized within a short timeframe compared to the production lifespan, typically one year for a product with a market life of two to three years. However, the adoption of state-of-the-art automation is often expensive and incurs additional costs due to the need for troubleshooting and specialized expertise in automation and machine learning. Consequently, there is a pressing need for streamlined and cost-effective automation workflows that rely on commercial off-the-shelf hardware and minimal expert intervention to meet the technical requirements. In this way, the full potential of automation methods can be realized.

Pose estimation is one critical aspect of assembly automation [1,2]. Machine learning (ML) techniques, and particularly convolutional neural networks (CNN), have revolutionized object detection and are also transforming pose estimation methods [3]. Despite the progress made by ML-based methods, new bottlenecks have emerged, which are now associated with the involvement of ML expertise and the need for extensive training data. State-of-the-art machine learning-based methods often suffer from poor performance when dealing with limited training data. Furthermore, their performance can be sensitive to network design decisions and hyperparameter choices. While these technologies offer new capabilities, their requirements hinder their widespread adoption in the industry.

This paper proposed a novel approach to pose estimation that prioritizes cost effectiveness in addition to accuracy. The method relies on stereo vision and can be easily set up using a variety of off-the-shelf or custom camera systems that meet the diverse requirements of assembly tasks on the production floor, such as specific designations of the field of view, working distance, and resolution. Although our approach benefits from deep learning, its performance is not heavily dependent on the quality of the deep learning module. We employed a deep neural network to primarily produce initial keypoint detections using limited training data. Subsequently, the preliminary keypoint detections are further enhanced through the application of auxiliary refinement processes. To accomplish this, we complemented the neural network detection with conventional feature extraction techniques. Furthermore, we incorporated information about the geometry of the mechanical parts of interest into the keypoint detection and pose estimation processes, assuming that these parts are rigid and that accurate knowledge of their design is available. These assumptions hold true for most parts found on assembly floors. Following pose estimation, nominal assembly trajectories were updated. A nominal trajectory is one that, given the reference position of all the components involved, can effectuate a successful assembly. While we describe our approach in the context of assembly automation, the techniques discussed here are general enough to be applicable to other aspects of manufacturing automation, such as in-situ inspection and error-proofing or to improve an existing pose estimation scheme.

Our pose estimation solution was based on keypoint detection, which centers on a deep neural network trained to detect a small number of keypoints on each mating assembly component. The method relies on stereo vision, which provides a lot of flexibility and removes the constraints and limitations of commercial technologies based on structured light. To address the challenges associated with adopting deep learning solutions, which include the need for machine learning expertise and large quantities of labeled training data, we developed a streamlined process that assumes neither of these requirements can be adequately met. The process begins with acquiring a small number of images of the assembly parts on the production floor and annotating them to train a deep learning architecture. We assumed that an automation technician with a basic understanding of the underlying deep learning process is responsible for setting up the training stage, possibly according to a simplified protocol that can be quickly developed and used for a variety of parts. The workflow, however, presumes that computer-aided design (CAD) models of the assembly parts or the precise relative positions of the keypoints of interest are available.

A deep network, when provided with insufficient training data and without the benefit of machine learning expertise, is likely to perform poorly and may not meet the requirements of the task at hand. In addition, the hardware flexibility associated with the adoption of stereo vision, comes at a cost—even with substantial amounts of training data and extensive effort on network design and tuning, meeting the accuracy requirements of object localization for assembly automation becomes challenging due to stereo vision’s sensitivity to disparity errors. It is worth noting that this is unlike mobile robotic or object grasping applications where the accuracy requirements for pose estimation are generally more relaxed and deep learning techniques have already demonstrated promising results [4,5]. In most industrial assembly tasks, the error should be limited to millimeter or even sub-millimeter scale for successful automation. Our study aims to achieve assembly-level accuracy while maintaining a practical approach suitable for implementation on the production floor, by eliminating the requirement for hyper-parameter optimization and minimizing the efforts involved in data collection and annotation when tailoring to new assembly tasks.

We proposed a Bayesian update method, which we refer to as Bayesian-Assisted Inference (BAI), to complement the deep network. We presumed that the network is able to identify a small subset of keypoints with reasonable accuracy and, despite its crude performance for the remaining keypoints, it can still offer a coarse a priori estimation of their pixel coordinates. Assuming the parts were rigid, we capitalized on our knowledge of their geometry to construct a set of likelihood functions that pinpoint the regions within an image where the remaining keypoints are expected to be located. This enabled us to generate a more precise posterior probability of keypoint locations. Finally, while the resulting posterior keypoints were still insufficient for disparity calculation and pose estimation with sufficient accuracy for assembly automation, we used conventional feature extraction techniques to find correspondences for the keypoints extracted through the BAI stage. These steps culminated in a pose estimation algorithm that is more tolerant of the deep network’s suboptimal performance and can be implemented quickly.

The contributions of this work can be summarized as follows: (a) Via a Bayesian updating framework, we present a novel and systematic method of incorporating our precise knowledge of part design into improving pose estimation accuracy. A significant implication of this approach is the potential for a marked reduction in data requirements, specifically within the realm of deep-learning-based pose estimation. This not only enhances efficiency but also advances the accessibility of such methods; (b) we demonstrate that traditional feature extraction techniques, such as Scale-Invariant Feature Transform (SIFT), when used in synergy with contemporary deep learning, can enhance the accuracy of pose estimation methods; (c) we provide a comprehensive ablation study to better understand the role of various components of our algorithm in improving accuracy or data efficiency; (d) we demonstrate the effectiveness of our algorithm on a challenging production floor assembly task. In particular, we applied our method to the assembly of a 14-point snap-fit dash trim on a Ford Mustang dashboard. The component features multiple challenges: it has a curved and complex geometry, lacks surface features, and is difficult to handle. Our algorithm exhibits effectiveness and simplicity in implementation, underscoring its potential for use in real-world applications.

The rest of the paper is structured as follows. Section 2 presents an overview of the past research on pose estimation. Section 3 discusses the proposed method including the pipeline and BAI. In Section 4, the experimental results are presented. Section 5 concludes the paper.

## 2. Related Work

### 2.1. Pose Estimation

Pose estimation is a crucial component of vision-based robotic assembly systems, involving the estimation of a target object’s rotation and translation relative to a reference. Structured light-based methods for pose estimation have been employed in various studies [6,7,8,9,10]. This technology necessitates the use of one or two camera systems equipped with an infrared (IR) projector, and relies on intricate pattern projection and matching algorithms. Such complexity renders custom implementations [7] challenging to achieve, particularly when attempting to satisfy the specific requirements (e.g., field of view) of each individual use case. This is especially problematic given the limited hardware specifications of existing off-the-shelf structured light systems, such as field-of-view, accuracy, and working distance, which make them unable to fulfill the diverse requirements of assembly applications. Moreover, pose estimation relying on structured light is susceptible to inaccuracies resulting from ambient illumination [11], as well as color and intensity distortions due to the absorption and reflectivity properties of objects in the scene [12].

Stereo vision [13,14] provides a convenient alternative to structured light methods, requiring only two regular cameras installed and calibrated on the same base platform. In a more general scheme, the cameras (possibly more than two) may be placed such that each captures a different view of the objects [15]. Stereo vision benefits from the availability of open-source software and extensive past research [16]. The simplicity of the setup allows for flexible selection of camera specifications that are most suitable for the given assembly task. Despite its advantages, the accuracy and reliability of conventional stereo vision techniques rely heavily on objects having distinct surface features [17], without which pose estimation through the resulting point cloud may fail. In many manufacturing applications, however, assembly parts have a plain finish and lack distinguishing surface features, which can complicate pose estimation through conventional means. Moreover, both stereo vision and structured light methods produce point cloud data that necessitate further processing, including the segmentation and their subsequent utilization for pose estimation [18].

Recent developments in 3D pose estimation have centered on deep learning methods. In end-to-end approaches, the 3D pose is directly predicted from an image [19,20] or an image pair [15]. Alternatively, in a process similar to what we adopted in this work, deep networks are utilized as the first step of two-stage approaches that first predict the 2D locations of specific keypoints on the object’s surface and then estimate the pose through the PnP algorithm [21,22,23].

### 2.2. Concurrent Object Detection and Pose Estimation

In recent years, methods that accomplish both object detection and pose estimation have gained popularity. Modern object detection models are already capable of accurately providing object bounding boxes or masks [24,25,26]. As an extension of the existing object detection or segmentation frameworks, the associated model structures are modified and the CNN-extracted features are reused as part of multi-task learning to concurrently perform object pose estimation [19,27,28,29,30]. However, these methods are heavily reliant on objects’ texture and context information and can be negatively affected by occlusion and cluttered backgrounds. Point-wise feature-based approaches [31,32,33] have gained popularity due to their better performance in such cases. These approaches focus on capturing local features and can more accurately estimate the pose of an object in 3D space. In general, the state-of-the-art machine learning-based techniques, including those based on point-wise features, can exhibit low generalizability and robustness issues [34] and therefore require large and diverse datasets for training. The main characteristics of some relevant works in the domain of 6D pose estimation are summarized in Table 1.

## 3. Proposed Technique

Our work focused on achieving pose estimation through the detection of a small set of keypoints on assembly parts using deep convolutional networks. After detecting these keypoints, they were further refined using a Bayesian-assisted inference technique. We began by providing a brief overview of our proposed approach, followed by a detailed explanation of its various stages.

### 3.1. Pipeline Summary

**Figure 1:**
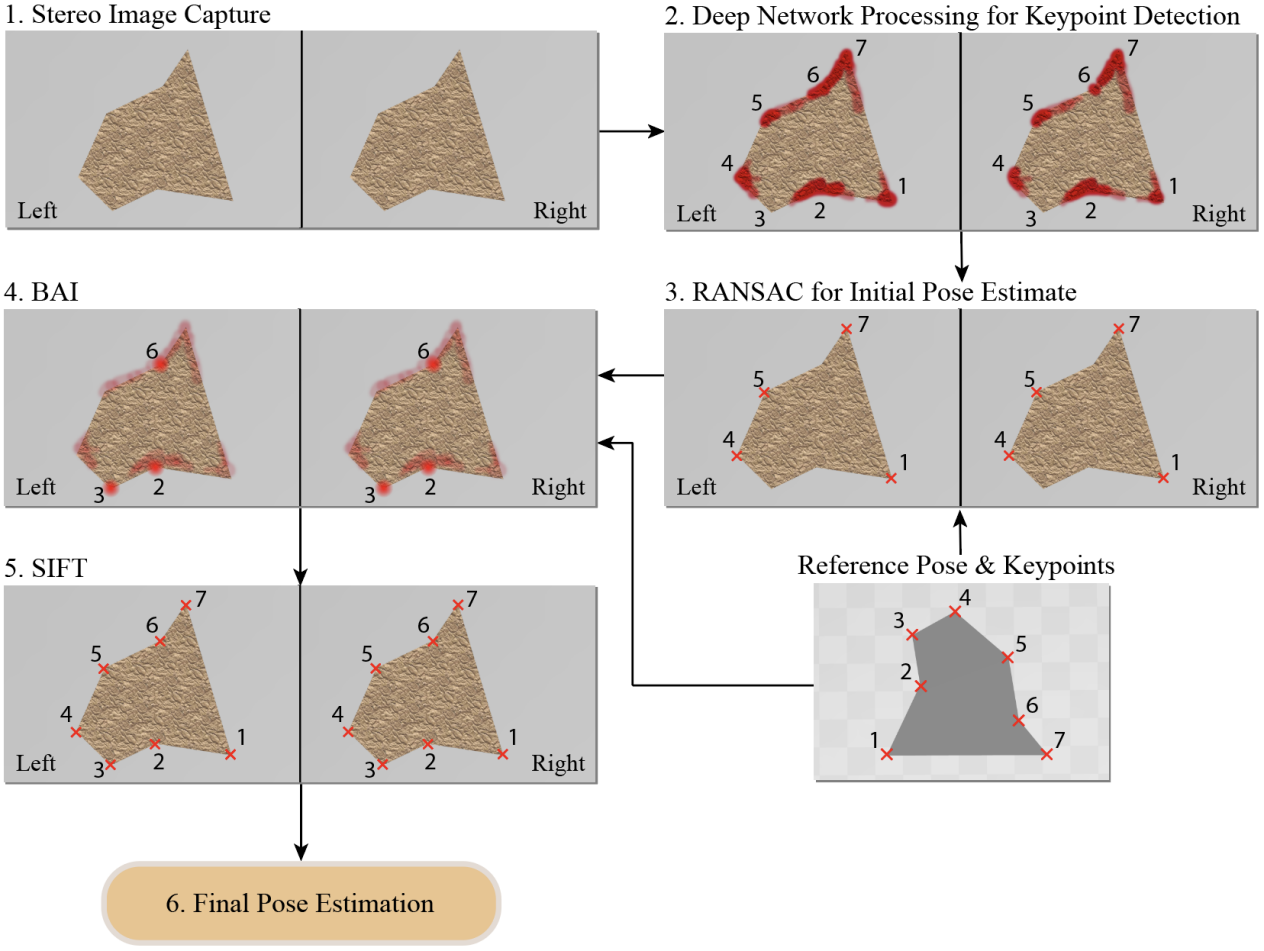
Keypoint detection and pose estimation pipeline consisting of 6 steps. (1) The stereo frames are captured through the camera; (2) frames pass through a keypoint detection network; (3) a RANSAC step identifies network output channels with higher quality and estimates an initial pose. In this schematic, keypoints 1, 4, 5, and 7 are detected as inliers by RANSAC; (4) the remainder of network channels (outliers) are updated through BAI using the part geometry. The associated keypoints are then extracted; (5) keypoint correspondences are obtained for all keypoints using SIFT; (6) a final more accurate pose is estimated.

Figure 1 demonstrates the pipeline for the proposed scheme and Algorithm 1 presents the associated pseudo code. The process starts with capturing the left and right stereo camera frames of the assembly components. Both frames pass through a deep convolutional network that outputs multiple heatmaps, one for each keypoint. These heatmaps are interpreted as 2D probability distributions of the keypoint pixel positions. Given the limited training data, and no or limited tailoring to the given assembly task, it is expected that the network performance be unacceptable for the majority of the output channels (heatmaps). As such, we assumed that only a small subset of keypoints will have heatmaps of sufficient quality to support obtaining an initial rough estimate of the pose. To identify the higher-quality heatmap channels, we relied on random sample consensus (RANSAC) [35]. This involves an optimization step that aims to find an initial rough estimate of a transformation matrix mapping the corresponding keypoints from a reference pose to their current position. Once the inliers are detected by RANSAC, the remaining extracted keypoints are considered outliers, and their corresponding heatmaps are only used as prior distributions of keypoint pixel positions. The estimated transformation matrix is then used to form a likelihood function for the outlier keypoints. The likelihood function is used to improve the priors (low-quality heatmaps) and form improved posterior distributions. We use these improved heatmaps to come up with a maximum a posteriori (MAP) estimate of the pixel positions.
**Algorithm 1** Keypoint detection and pose estimation pipeline.**Inputs:**    Image pair from stereo camera: $img_{l}$, $img_{r}$    Locations of *N* reference keypoints $K=[k_{1},k_{2},\ldots ,k_{N}]$    Minimum number of valid keypoints L≥4**Outputs:** Transformation matrix $M={\hat{M}}^{*}$1:Grab a pair of stereo images $\left\{{img}_{l},{img}_{r}\right\}$2:Pass through CNN, obtain keypoint heatmaps H=CNN(img).3:**for** iteration t∈ 1 to *T* **do**4:    1. Choose a random subset of heatmaps and extract the 2D keypoint positions.5:    2. Obtain the transformation matrix, $\hat{M}$, according to Equation (1).6:    3. Determine the inliers and outliers based on the projection error.7:    4. Store $\hat{M}$ if there are more inliers than the latest best projection.8:**end for**9:Return $\hat{M}$ and the indices of the inlier and outlier keypoints ($S_{I}$ and $S_{O}$).10:Form likelihood functions for all keypoints in $S_{O}$ per Equation (2).11:Form priors for keypoints in $S_{O}$ ($P_{n}^{H}=H_{n}$).12:Form posterior probabilities for keypoint in $S_{O}$ per Equation (3).13:Find the MAP estimate of pixel positions for kepoints in $S_{O}$ per Equation (4).14:Use SIFT to find correspondences for all keypoints in both $img_{l}$, and $img_{r}$.15:Take average of disparity pairs for each keypoint.16:Find the final transformation, $M={\hat{M}}^{*}$, through the PnP algorithm (or per Equation (1)).

**Figure 2:**
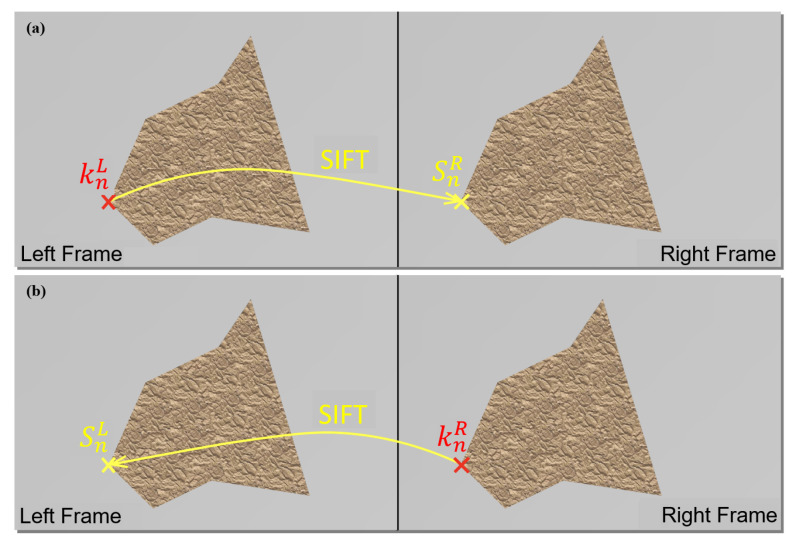
Use of SIFT to improve disparity estimate. Use the keypoint position in the (**a**) left/(**b**) right image, ($k_{n}^{L}$, $k_{n}^{R}$) as a reference to find its SIFT correspondence position in the (**a**) right/(**b**) left frame, ($S_{n}^{R}$, $S_{n}^{L}$). We take an average of the resulting two disparities.

Even for a high-quality posterior, the accuracy of keypoint positions are generally insufficient for pose estimation in assembly automation. This is a consequence of the sensitivity of stereo vision to disparity errors, inability of human annotators to achieve perfect annotation quality, in addition to the sub-optimal performance of the network. As such, for point correspondences, we relied on scale-invariant feature transform (SIFT) [36]. SIFT was applied to the extracted keypoints in both the left and right frames independently, to find an accurate correspondence for each keypoint. SIFT is an accurate tool for correspondence discovery but suffers from computational complexity [37]. However, here we could limit the SIFT search area to the regions surrounding the obtained keypoints. This dramatically reduces the computational load and enables real-time implementation. Following these steps, we will have two pairs of correspondences leading to two disparity values (Figure 2). Assuming that the estimated disparity was contaminated by a zero-mean noise, we took an average of the two disparities, thereby theoretically reducing the estimation variance by a factor of 2. The averaged disparities were used to make a final, more accurate estimate of the pose.

### 3.2. Bayesian-Assisted Inference (BAI)

Training a deep neural network with a generic backbone, limited data, and suboptimal hyperparameters in the absence of ML expertise can result in poor performance. This is particularly true for keypoint detection, as the visual features of different keypoints can be highly similar, leading the network to identify multiple islands of high probability pixels for each keypoint and decreasing its overall accuracy. The conventional approach for a poorly performing network is to collect more training data, use data augmentation strategies, optimize the network architecture, and fine-tune the hyperparameters. This process is iterative and can be expensive, requiring expert knowledge. However, we argue that it is possible to improve the network’s performance without collecting additional data or training by adopting alternative methods.

We proposed two main assumptions to improve network performance without additional data or training: (1) the network can detect at least four keypoints with reasonable accuracy, which forms an over-determined system of equations and yields an initial but imprecise estimate of the pose; (2) we assumed that assembly parts behave rigidly, meaning that the relative positions of the keypoints of interest remain unchanged and that we have access to their accurate CAD models. An initial transformation matrix was obtained based on the first assumption and served as a starting point for refining the pose estimate. The second assumption, together with the initial transformation matrix, enabled the formation of likelihood functions that describe the probable positions of poorly detected keypoints, thereby enhancing the accuracy of the final transformation matrix within a Bayesian updating framework.

**Figure 3:**
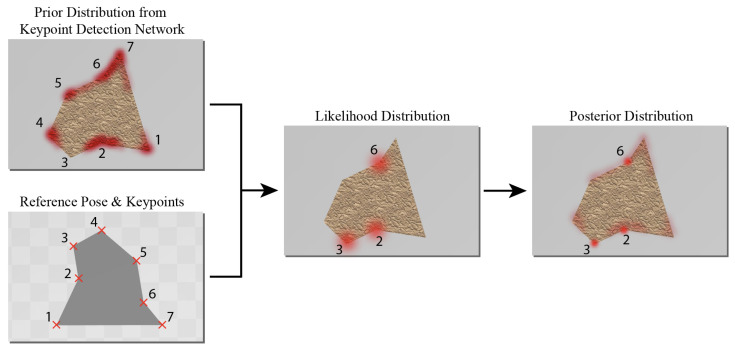
Bayesian-assisted inference: In this 2D example, keypoints 1, 4, 5, and 7 are the selected keypoint subsets to infer the likelihood function for the remaining keypoints. Thus, the location of the outliers (keypoints 2, 3, and 6) are corrected in the posterior.

This is conceptually demonstrated in Figure 3, in which the objective was to identify keypoints associated with the corners of a 2D polygon. In this example 2D case, having access to approximate positions of a subset of keypoints, one can identify the likely positions for the remaining keypoints and form an improved posterior for each. To paint a more intuitive picture of the proposed concept, consider a case where, as part of a human joint detection task, only feet and waist are identified. Given our knowledge of the human anatomy, in such a case, one can come up with the likely regions where the knees may be located. Analogous to this example, here, by utilizing our initial estimate of the transformation matrix, we incorporated our knowledge of the part design into the formation of likelihood functions. This is further elaborated in the following.

#### 3.2.1. Preliminaries

To transform the 3D position, $X_{n}$, of the $n^{\mathrm{th}}$ reference keypoint (n∈{1,...,N}) to its new position, $X_{n}^{\prime }$, we canwrite:

$$X_{n}^{\prime }=MX_{n},$$

where $X_{n}={\left[x_{n}\;y_{n}\;z_{n}\;1\right]}^{T}$, $X_{n}^{\prime }={\left[x_{n}^{\prime }\;y_{n}^{\prime }\;z_{n}^{\prime }\;1\right]}^{T}$, and $M=\left[\begin{array}{cc} R & T\\ 0 & 1 \end{array}\right]$ is the corresponding transformation matrix. Here, *R* is a 3×3 rotation matrix, *T* is a 3×1 translation vector, and $X_{n}$ and $X_{n}^{\prime }$ are both in homogeneous coordinates. In addition, given the camera calibration matrix, one can project a 3D point, $X_{n}$, onto the image coordinates $k_{n}=\left[x_{n}^{I},y_{n}^{I}\right]$, where $x_{n}^{I}$ and $y_{n}^{I}$ are lateral and vertical pixel positions, respectively.

We denote the probability of the $n^{\mathrm{th}}$ keypoint residing at the 2D pixel position $k_{n}$ as $P_{n}\left(k_{n}\right)$. We denote the corresponding prior and posterior probabilities as $P_{n}^{H}\left(k_{n}\right)$ and $P_{n}^{P}\left(k_{n}\right)$, respectively.

The output of the keypoint detection deep network includes *N* channels, each representing a heatmap, $H_{n}$, for each of the keypoint locations. The heatmap coordinates associated with larger values are indicative of more likely pixel positions for the corresponding keypoint.

#### 3.2.2. Initial Estimation of the Transformation Matrix, *M*

We assumed that the performance of the network is reasonable for a subset of keypoints, denoted as $S_{I}$, with a size L≥4. The pixel position of each of these keypoints can be extracted from the network heatmaps as:

$${\hat{k}}_{n}=\arg\max_{k}H_{n}\left(k\right),$$

where $k_{n}$ and *k* are 2D pixel positions. Given the left- and right-frame pixel positions for each keypoint, one can calculate disparity and subsequently find an estimated 3D position, ${\tilde{X}}_{n}$, for each keypoint in $S_{I}$. An initial, although rough, estimate of the transformation matrix *M* can then be found:

(1) $$\begin{array}{cc} R(M) & =\left|\right|\tilde{X}-MX\left|\right|,\\ \hat{M} & =\arg\min_{M}R\left(M\right), \end{array}$$

where $X=[X_{l}]$ and $\tilde{X}=\left[{\tilde{X}}_{l}\right]$, ($l\in S_{I}$) represent the 3D positions of the reference and estimated keypoints.

In the above equations, it is assumed that we have access to $S_{I}$, meaning we know which output channels of the network have higher quality to support an initial estimate of *M*. However, in practice, we do not have access to this information. To identify this subset of the network output channels, we used RANSAC (RANdom SAmple Consensus) [35] for outlier/inlier detection as part of the optimization of Equation (1)). The minimum number of inliers, *L*, was treated as a hyperparameter and, as noted earlier, was assumed to be ≥4. The identified inliers constitute the set $S_{I}$ and the optimal result, $\hat{M}$, is taken as one initial measurement of the transformation matrix. The remaining, N−L, keypoints (outliers), constitute the set, $S_{O}$, for which we consider the network generated heatmaps as prior probabilities of their pixel positions, i.e., $P_{n}^{H}=H_{n}$.

#### 3.2.3. Likelihood and Posterior

Next, we will focus on the subset of keypoints, denoted by $X_{m}$ for $m\in S_{O}$, that are considered outliers due to their poor network output quality according to the RANSAC algorithm. For a given initial estimate, $\hat{M}$, of the transformation matrix, we can write:

$$\begin{array}{c} {\hat{X}}_{m}^{\prime }=\hat{M}X_{m},\\ {\hat{F}}_{m}=P{\hat{X}}_{m}^{\prime }, \end{array}$$

where *P* is the camera calibration matrix, ${\hat{F}}_{m}={\left[{\hat{k}}_{m}\;{\hat{z}}_{m}\right]}^{T}$ and ${\hat{k}}_{m}={\left[{\hat{x}}_{m}^{I}\;{\hat{y}}_{m}^{I}\right]}^{T}$.

We assume that, for any given pixel position $k_{m}$ of the outlier keypoint *m*, the estimate ${\hat{k}}_{m}$ is more likely to be located in close proximity to $k_{m}$, and the likelihood of the estimate decreases as it deviates from this position. We consider a Gaussian form for the likelihood function:

(2) $$P^{L}\left({\hat{k}}_{m}|k_{m}\right)=\frac{1}{2\pi \sqrt{\left|\Sigma \right|}}e^{-\frac{1}{2}\left(k_{m}-{\hat{k}}_{m}\right)\Sigma^{-1}\left(k_{m}-{\hat{k}}_{m}\right)},$$

where $P^{L}$ is the likelihood function, Σ=σI, *I* is a 2×2 identity matrix and σ is treated as a hyperparameter. Given the prior (network heatmap) and the likelihood function, the posterior probability can be written as:

(3) $$P^{P}\left(k_{m}|{\hat{k}}_{m}\right)\propto P^{H}\left(k_{m}\right)P^{L}\left({\hat{k}}_{m}|k_{m}\right).$$

This formulation provides us with a systematic method of incorporating our accurate knowledge of the part geometry in improving the network-generated heatmaps. We then rely on the maximum a posteriori (MAP) estimate of the keypoints pixel positions, which is expected to be superior to that solely based on the network priors:

(4) $${\tilde{k}}_{m}=\arg\max_{k_{m}}P_{P}\left(k_{m}|{\hat{k}}_{m}\right),$$

where, as before, keypoint *m* belongs to the outliers. By integrating the posterior-based 2D keypoint positions with the 2D positions of the inlier keypoints directly obtained from the network, we can derive a more accurate estimate of the transformation matrix, *M*.

As noted earlier, even with the improved posteriors, we may not be able to obtain an accurate disparity estimate. As such, we relied on SIFT to find the correspondences (see Section 3.1 and Figure 2), and calculated disparity for each keypoint. This was then used to estimate the 3D positions of all keypoints and ultimately a final, more accurate ${\hat{M}}^{*}$ according to Equation (1)). To find the final ${\hat{M}}^{*}$, we included all the keypoints in $S_{I}\cup S_{O}$. We provide the complete pose estimation process including the BAI step in Algorithm 1.

## 4. Experimental Evaluation

### 4.1. Experimental Setup and Training Data

**Figure 4:**
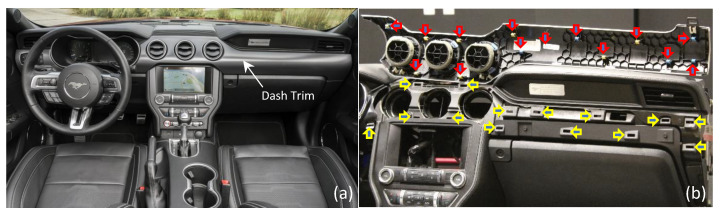
(**a**) Assembly of dash trim onto Mustang dashboard. (**b**)The dash trim is thin and slender with a large aspect ratio, making it rather difficult to handle. Trim features 14 locking pins on the back (red arrows) that need to mate with 14 holes on the dashboard (yellow arrows).

As an example application, here we studied the assembly of a Mustang dash trim onto the dashboard (Figure 4). The dash trim has a complex and curved geometry, and its surface has no visually distinct features, which makes it a very challenging candidate for pose estimation. The dash trim’s slender design with a large aspect ratio also makes its handling and assembly difficult. It features 14 pins with associated locking keys on the back of the dash trim that need to properly mate with 14 holes on the dashboard for a successful assembly (Figure 4b). This assembly task relies on the operator’s experience and training, and damage during assembly is common, leading to a large number of scrap parts. As such, we also found this a worthwhile and challenging candidate for an assembly automation case study. In the remainder of this section, we focus on the implementation and evaluation of the proposed algorithm through this assembly task, including a comprehensive ablation study to better understand the behavior of various components of our method.

**Figure 5:**
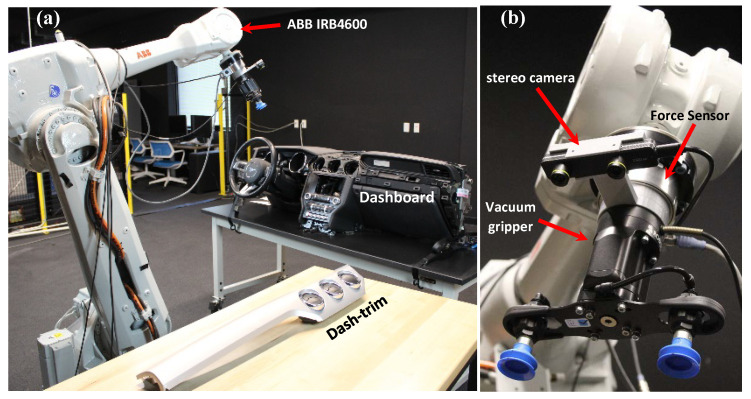
(**a**) Assembly setup: an ABB IRB 4600 industrial robot is used to pick up a dash trim and install on a dashboard, (**b**) The end effector including a force sensor, stereo camera, and vacuum gripper.

Figure 5 shows the experimental setup. An ABB IRB 4600 industrial robot was used to automate the assembly task. The figure also demonstrates the robot end-effector, including an EPick Robotiq vacuum gripper, a ZED-mini stereo camera and an ATI IP65 Omega85 force transducer. The force sensor was only used for safety purposes and emergency stop, and to ensure that the parts are not exposed to extreme forces causing damage. The task involves localization of the dash trim, placed at a random location on a table. To ensure successful execution of the assembly task, it is crucial to minimize the pose estimation error. This is necessary to achieve precise manipulation of the dash trim using the vacuum gripper, as well as for accurately aligning the pins and holes during the assembly process.

To create a quality training dataset, it is important to include a wide range of images depicting the mating parts. One approach is to position the parts in random locations and orientations that closely resemble their arrangement on the production floor. For each configuration, images can be captured and annotated. However, this process is time-consuming and labor-intensive. Moreover, conducting extensive development activities directly on an active production floor is often impractical. Consequently, these activities are typically performed in a replicated robotic assembly setup within a controlled laboratory environment before implementation which can result in increased costs and potential disparities between the train and test conditions.

To optimize the data collection process on the production floor, we employed an eye-in-hand robot arm. This robot arm not only captures images from various perspectives to collect the training data but also performs the final assembly task. By maneuvering the camera around the assembly parts, images with varying perspectives can be captured, without having to alter the pose of the parts. Furthermore, we kept the number of annotated images at a manageable level, typically limited to a few hundred, and restricted the number of keypoints to less than 10 per object. In our specific experiment with dash trim, we utilized a total of 277 samples and seven keypoints.

Although this data generation method is streamlined, it has limitations in capturing intricate variations associated with shadows, background features, and relative object positions. Consequently, the network performance may be affected. In the following, we demonstrate that our proposed approach enhances the network’s performance, even when trained on limited and relatively low-quality data, and subsequently improves pose estimation.

**Figure 6:**
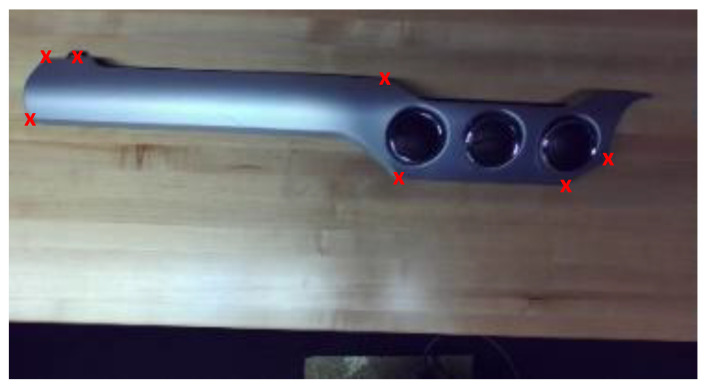
Example annotated image of dash trim. The annotated keypoints are shown with red crosses.

Figure 6 shows an example of a dash trim image with the corresponding annotations as red crosses. As shown in this figure, the dash trim has a plain surface with no distinct visual features. Hence, most of the keypoints were selected on the boundary with sharp geometries. It was noted that increasing the number of keypoints can potentially improve pose estimation accuracy. For a component example such as the one shown in Figure 6, given its curved and featureless surface, there are not too many keypoint options. It is also important to note that it is possible to select keypoints in such a way that they span multiple camera viewpoints. In that case, it is necessary to adopt registration schemes [10], which may complicate the implementation and, depending on the registration performance, adversely affect pose estimation accuracy. As such, when possible, it is preferred to select the keypoints on a single view-point.

### 4.2. Network and Training

#### 4.2.1. Network Architecture

**Figure A1:**
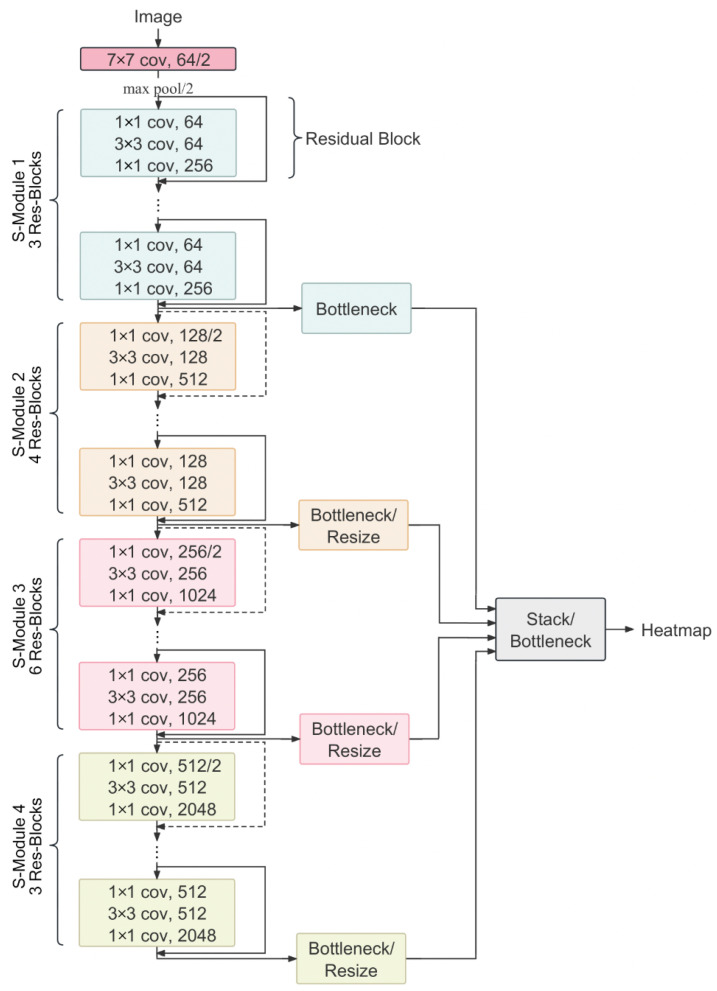
Keypoint detection network architecture. Residual blocks belonging to the same S-Module and the corresponding bottleneck blocks are shown in the same color. Within an S-Module, only the first and the last residual blocks are shown in the figure, and the rest are indicated by ellipses. The arrows directly connecting the input and the output of residual blocks are named shortcut connections that perform identity mapping from the input to the output for residual learning. Among the shortcut connection arrows, the dotted ones indicate that dimension increase is also performed during shortcuts.

For keypoint detection, we relied on a deep CNN architecture as shown in Figure A1 in Appendix A. This architecture was inspired by earlier works in human joint detection such as a cascaded pyramid network [38]. The feature extractor has 50 layers [39] and starts with a convolutional layer featuring 7×7 kernels and a stride of two followed by a max pooling layer to reduce the input image size (1242×2208) by a factor of 2 along each dimension to 621×1104. The result then passes through four consecutive modules (noted as S-module in Figure A1), each featuring multiple residual blocks [39]. The S-modules together form a feature pyramid network [40] with four outputs, each featuring a different scale. The output of the S-modules passes through a bottleneck to adjust the number of channels to match the number of keypoints and is then resized to match the size of the first S-module output (i.e., 311×552). As such, the output of the bottlenecks is 311×552×N, where *N* is the number of keypoints to be detected by the network. The bottleneck outputs are stacked up to form a 311×552×4N output, which is further passed through a final bottleneck stage to reduce the number of channels back to *N* (i.e., 311×552×N). In other words, each channel on the output creates a heatmap of the potential positions of one of the *N* keypoints. The final bottleneck combines the features extracted at various scales; some at lower level S-modules focused locally around the keypoints of interest, while others at higher level S-modules looking at a larger picture, taking into account the contextual information, including the relative position of various keypoints. The output of each residual network is batch normalized prior to the nonlinear activation, which also helps with regularization. An exponential linear unit (ELU) activation is used in each residual unit.

#### 4.2.2. Training

Throughout the training process, we penalized non-zero values on the outputs of the four bottlenecks and the final output of the network, with the exception of the regions surrounding the keypoints of interest. In this form, the loss function is a sum of five terms (associated with the final output heatmap and the outputs of the four bottlenecks). Each loss term is defined as the root mean square (RMS) error between the corresponding output and the ground truth heatmap. The ground truth heatmap is formed by placing a Gaussian blur [38] with a variance of 10 pixels at the location of each keypoint, forming a 310×552×N target output. The variance of the Gaussian blur is a hyperparameter which is tuned to reflect the human annotation uncertainty and the image resolution and quality limitations. Consistent and accurate annotation supports the generation of a sharper target heatmap, resulting in better keypoint detection. Further insights into the loss function and its components can be found in [38].

An Adam optimizer [41] with an initial learning rate of $5\times 10^{-4}$ was used throughout the training. During inference, the 2D pixel location of the maximum heatmap value in each of the *N* channels of the network output was used as the position of the corresponding keypoint. Since the size of each output channel (heatmap) was smaller than the original image size, the coordinates of the maximum heatmap values were scaled up to determine the pixel position of the keypoints on the input image. The model was trained for 300 epochs, and the best validation model was used to run the tests in Section 4.3.

### 4.3. Results

**Figure 7:**
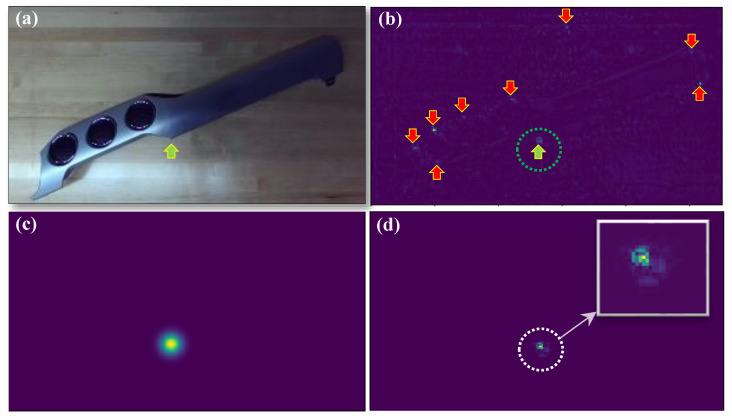
(**a**) A test dash trim image with one of the keypoints denoted by the green arrow. (**b**) Network heatmap output associated with the keypoint of part (**a**); multiple regions on the heatmap demonstrate strong excitation, indicative of the network confusion (red arrows). The green dotted circle represents the neighborhood of the local maximum corresponding to the accurate keypoint position. (**c**) Likelihood function obtained based on Equation (2). (**d**) Updated network output (posterior) using BAI (Equation (3)).

Figure 7a depicts an example test image of the dash trim and Figure 7b illustrates the corresponding heatmap generated by the network for a single keypoint. The keypoint of interest, indicated by a green arrow in both figures, is further distinguished by a green dotted circle in Figure 7b. As can be observed, in addition to the immediate vicinity of the keypoint location, the heatmap also displays large values in various other regions. In fact, in this case, the location of the heatmap maximum value occurs far from the true position. While the heatmap global maxima is misleading, further examination reveals the presence of a relatively accurate local maxima, indicative of the true keypoint position in the immediate vicinity of the ground truth. This underscores the importance of incorporating seemingly low-quality heatmaps as priors when estimating the position of the keypoints. The proposed BAI enabled us to take into account the part geometry to improve this heatmap. Figure 7c,d shows the likelihood and the posterior heatmaps, respectively. The posterior heatmap exhibits excitation exclusively in the region of the image where the correct keypoint is located. The final position of the keypoint can be extracted as the 2D location of the maximum posterior probability. The same process was applied to the remaining keypoints and the resulting updated heatmaps were used to extract more reliable pixel positions. These were then used in the calculation of a final, more accurate pose. The execution time of the algorithm from image acquisition to pose estimation is about 315 msec on an AMD Threadripper 3970X CPU and an NVIDIA RTX A6000 GPU, which makes it suitable for real-time implementation.

**Figure 8:**
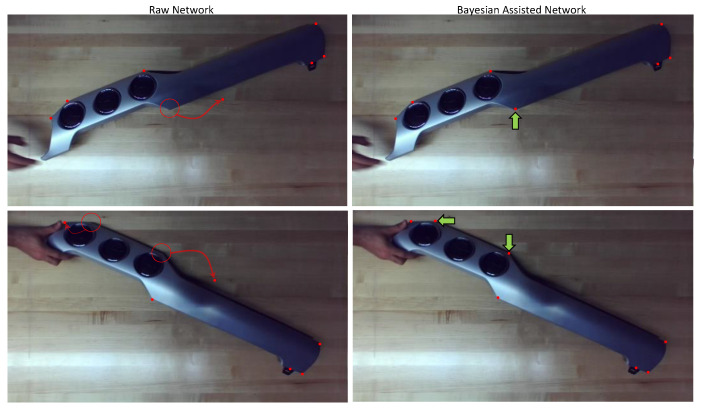
(**Left**) Identification of keypoints (red dots) using the raw trained network without BAI. Location of the missed keypoints are denoted by red circles. Misplaced keypoints are denoted by the red arrows. (**Right**) Improved results with BAI; all keypoints are detected correctly (red dots and green arrows).

Figure 8 shows two examples (left-hand side) in which certain keypoints were misplaced (red arrows) when the raw trained network was used. After applying Bayesian updating and integration (BAI), all previously mislocated keypoints were correctly detected, as shown on the right-hand side of Figure 8.

In order to assess the pose estimation performance of the proposed method, the position and orientation of the dashtrim relative to the camera were varied, and the resulting pose was estimated. To evaluate the accuracy of the pose estimation, access to the ground truth pose was required, which was not readily available. Therefore, instead of moving the part itself, we adjusted the camera position and recorded the corresponding robot pose for each frame.

We evaluated the performance of our method quantitatively. We further conducted a series of ablation studies to evaluate the impact of various algorithm components and parameters on the overall accuracy and robustness of the system. The results of these studies are summarized in Table 2 and Table 3. In addition to our proposed method (1) (BAI + SIFT), we evaluated the pose estimation accuracy when the SIFT module was disabled or when only the SIFT module was active while BAI was disabled. Table 2 includes the results for two different sizes of training data and summarizes all the ablation results against the baseline where neither SIFT nor BAI are applied (first row). In all these studies, 20% of the data is used as validation and the rest for training. For more reliable evaluation, we used a larger set including 511 annotated images for testing. As shown in Table 2, for our proposed approach (BAI + SIFT) using 100% of the available data (277 images), the mean position estimation error was at 2.8 mm with a standard deviation (SD) of 1.6 mm. The mean angular rotation estimation error was 0.08(SD = 0.06). These results meet our requirements for the automation of the given assembly task. The baseline performance shows a positioning error of 6.4 mm (SD = 5.7 mm) showing a three-fold increase compared to the proposed BAI with SIFT.

To evaluate the performance loss due to further reduction in data size, we trained a second model, using only 20% of the 277 annotated images, i.e., using 55 total annotated images (44 for training, 11 for validation). Note that this is considered an extremely small set for a network architecture of such capacity (with 50 layers and a total of 23.5 million trainable parameters) and can easily lead to overfitting. The baseline positioning error in this case was 16.1 mm (SD = 16.8 mm). With the adoption of our proposed BAI + SIFT, the positioning error reduces to 5.8 mm (SD = 2.7 mm), showing a three-fold reduction in mean error and a six-fold reduction in standard deviation. Hence, the network’s performance was improved to a level that is superior to the baseline case even when using the complete data (277 images), i.e., trained on a dataset five times larger. The effect of Bayesian assistance on rotation estimation, although noticeable, is not as dramatic, which may be attributed to the long and slender geometry of the dash trim, which facilitates angle estimation even for erroneous keypoint estimates. Table 2 also shows the rest of our ablation results, demonstrating the importance of SIFT and the synergy between SIFT and BAI.

We further evaluated the effect of disparity averaging. The results are listed in Table 3. As shown in this table, for both training data sizes, averaging plays an important role in reducing error and its standard deviation. Nevertheless, the difference in performance is not noticeable between the two cases where either the disparity (AD) or the resulting 3D estimates (AE) are averaged.

This table also highlights the low accuracy of the initial pose obtained through the RANSAC step (IE), where only the higher quality channels of the network are used. According to this result, by improving the outlier channels of the network and integrating them into the estimation process, the error mean/SD reduces from 16.31 mm/3.74 mm to 2.89 mm/1.52 mm. It is worth highlighting that the dataset of dash trim images consisted of seven keypoints, with four keypoints being utilized in the RANSAC estimation process. This left only three outliers for further enhancement through BAI. The results demonstrated a notable improvement in the overall estimation accuracy, even with such small number of outliers. It is reasonable to anticipate that, as the total number of keypoints, and potentially the total number of outliers, increase, the proposed method will become even more effective in enhancing the accuracy and robustness of the estimation. This is because with a larger number of keypoints and outliers, the inlier set has the potential to experience a more significant increase in size, leading to improved estimation performance.

Finally, we assessed the performance of the proposed method when implemented as part of an assembly task. To evaluate this, we pre-programmed the robot with an optimal trajectory for pick-up and assembly given the exact positions and orientations of the dash trim and dashboard. The dashboard pose was assumed to be available since it was mounted at multiple pre-defined points on a standard table on the production floor. As the pose of the dash trim deviates from its assumed location and orientation, the pick-up and assembly trajectory is dynamically adjusted. The assembly process was initiated by randomly placing the dash trim on a table, similar to the process on the production line. The camera was then repositioned to capture the table area within its field of view and estimate the new pose. Subsequently, the nominal trajectory was updated and the assembly task was executed. An illustrative video showcasing the assembly process was included in the Supplementary Materials.

## 5. Conclusions

In this paper, we introduced a streamlined vision-based pose estimation method that caters specifically to the constraints of production floors. Our approach involves training a deep neural network to detect the pixel positions of a small number of keypoints on the mechanical part of interest. To enhance the performance of the keypoint detection network, we have outlined a systematic method of incorporating precise part geometry information into the keypoint detection process.

The proposed method was formulated within a Bayesian updating framework, where we exploited the assumption of rigidity in the mechanical parts and the availability of accurate design information. We employed RANSAC to identify a subset of inlier network output channels, which provides an initial estimate of the part’s pose. The remaining outlier network channels are treated as priors within the Bayesian updating context. By utilizing the part geometry, we generated likelihood functions that contribute to an improved posterior probability distribution, enabling a more precise determination of the outlier keypoint locations. Subsequently, Maximum a Posteriori (MAP) estimates of the outlier keypoint positions, along with the initial inlier keypoint positions, were employed to calculate the final pose of the component. We have conducted a comprehensive evaluation of our proposed method, including quantitative assessments and ablation studies on a challenging component chosen from the Mustang assembly floor featuring complex geometry and a plain surface devoid of any visual features. We successfully demonstrated its effectiveness through the assembly of a dash trim on the dashboard. By presenting a systematic approach to incorporating our accurate knowledge of part geometry into pose estimation, showing its effect on improving the accuracy and data efficiency, and demonstrating the potential synergy between conventional feature extraction tools and contemporary deep learning, our research contributes to advancing the field of vision-based pose estimation in production floor settings.

Several future directions for this research can be explored. To further enhance the ease of adoption, it is advantageous to explore methods for automating the selection and annotation of keypoints. One potential approach is to generate synthetic photorealistic renderings of the objects of interest, enabling automated generation of annotated images. This eliminates human error and further reduces the cost of data generation for network training. Moreover, it is important to acknowledge the absence of benchmarks with diverse sets of mechanical components commonly found on production floors, along with their corresponding CAD models. These benchmarks should extend beyond bin-picking applications and, while addressing the accuracy requirements of assembly automation, should promote data-efficient, easy-to-implement, and robust 6D pose estimation technologies that can be utilized and maintained without machine learning or computer vision expertise. By doing so, we can encourage research that places a greater emphasis on practical considerations and is more likely to be adopted on production floors. Once such a benchmark becomes available, conducting a comprehensive ablation study on existing 6D pose estimation techniques would be invaluable. This will deepen our understanding of the strengths and weaknesses of different components in the state-of-the-art methods and potentially lead to hybrid approaches that better leverage the additional information available on the production floor.

## Figures and Tables

**Table 1:** A summary of relevant 6D pose estimation methods. End-to-end: fully ML-based method directly generates 6D pose. Generic: not part specific. Assem. prec.: assembly-level precision. Data efficient: designed with data efficiency as a constraint. Prod. level: designed with an emphasis on production floor requirements. Once deployed, can be maintained and adapted to new assembly tasks without the need for machine learning or computer vision expertise. Geom. info: can use geometric design data. CAD model: relies on CAD models. Object + pose: simultaneous object detection and pose estimation. Point cloud: reliant on dense point cloud measurements of objects. Opt: optional.

Attribute	Gen6D [19]	SSD6D [27]	PoseCNN [28]	ES6D [31]	Multi-v [15]	PVN3D [32]	DenseFusion [33]	BAI + SIFT
End-to-end	✕	√	√	√	√	✕	√	✕
Generic	√	✕	✕	✕	✕	✕	✕	✕
Assem. prec.	✕	✕	✕	✕	✕	✕	✕	√
Data efficient	✕	✕	✕	✕	✕	✕	✕	√
Prod. level	✕	✕	✕	✕	✕	✕	✕	√
Geom. info	✕	✕	✕	√	✕	√	√	√
CAD model	✕	✕	✕	√	✕	√	√	Opt
Obj + pose	✕	√	√	√	√	✕	√	✕
Point cloud	✕	√	√	√	✕	√	√	✕

**Table 2:** Displacement and rotation errors of the models trained on 20% and 100% of the available data. The two models were tested with and without BAI or SIFT. Results for the complete method are shown in bold.

Train Set	BAI	SIFT	Displacement (mm)		Rotation (°)	
			Mean	SD	Mean	SD
100%	-	-	6.43	5.57	0.09	0.11
	-	√	4.45	4.7	0.08	0.15
	√	-	6.02	4.1	0.09	0.07
	√	√	**2.87**	**1.63**	**0.08**	**0.06**
20%	-	-	16.11	16.86	0.57	0.53
	-	√	12.09	15.10	0.46	0.44
	√	-	9.33	5.09	0.56	0.42
	√	√	**5.89**	**2.77**	**0.52**	**0.36**

**Table 3:** Performance results on the 100% data for averaged disparity (AD), averaged 3D-estimate (AE), and no averaging, where one of the two disparity values were randomly selected (random disparity or RD). The IE (initial estimate) demonstrates the poor accuracy of the initial pose estimation based on RANSAC.

Train Set	Setting	Displacement (mm)		Rotation (°)	
		Mean	SD	Mean	SD
100%	AD	2.89	1.52	0.08	0.06
	AE	2.87	1.63	0.08	0.06
	RD	5.32	4.09	0.22	0.07
	IE	16.31	3.74	1.66	0.29
20%	AD	5.87	2.78	0.52	0.37
	AE	5.89	2.77	0.52	0.36
	RD	8.16	4.55	0.54	0.39

## Data Availability

The data utilized in this study is proprietary and its availability is restricted.

## Supplementary Materials

The following supporting information can be downloaded at: https://www.mdpi.com/article/10.3390/s23136107/s1, Video S1: Demonstration of the automated assembly process for a 14-point snap-fit dash trim onto a Ford Mustang dashboard based on the proposed pose estimation.

## Abbreviations

The following abbreviations are used in this manuscript:

2D	Two-dimensional
3D	Three-dimensional
BAI	Bayesian-assisted inference
CAD	Computer-aided design
CNN	Convolutional neural network
ELU	Exponential linear unit
IR	Infrared
MAP	Maximum a posteriori
PnP	Perspective-n-Point
RMS	Root mean square
SIFT	Scale-invariant feature transform

## Appendix A

Figure A1 shows the network architecture for keypoint detection. Table A1 lists the number of trainable parameters for various modules in the model.

**Table A1:** Number of trainable parameters of each modules in the architecture.

		Number of Trainable Parameters
Initial convolutional layer		9356
S-Module 1	Residual block 1	75,008
	Residual block 2	70,400
	Residual block 3	70,400
S-Module 2	Residual block 1	379,392
	Residual block 2	280,064
	Residual block 3	280,064
	Residual block 4	280,064
S-Module 3	Residual block 1	1,512,448
	Residual block 2	1,117,184
	Residual block 3	1,117,184
	Residual block 4	1,117,184
	Residual block 5	1,117,184
	Residual block 6	1,117,184
S-Module 4	Residual block 1	6,039,552
	Residual block 2	4,462,592
	Residual block 3	4,462,592
Bottleneck 1		1799
Bottleneck 2		3591
Bottleneck 3		7175
Bottleneck 4		14,343
Final bottleneck 1		203
Total number		23,535,143
